# The Prevalence and Incidence of Depression among Visually Impaired People: A Systematic Review and Meta-Analysis Protocol

**DOI:** 10.12688/f1000research.166020.1

**Published:** 2025-07-04

**Authors:** Pankaew Tantirattanakulchai, Nuchanad Hounnaklang, Nanda Win, Suchon Tepjan, Pear Ferreira Pongsachareonnont

**Affiliations:** 1 Department of Health Education and Behavioral Sciences, Faculty of Public Health, Mahidol University, Ratchathewi, Bangkok, 10400, Thailand; 2 College of Public Health Sciences, Chulalongkorn University, Pathumwan, Bangkok, 10330, Thailand; 3 VOICES-Thailand Foundation, Chiang Mai, Chiang Mai, 50100, Thailand; 4 Department of Ophthalmology, Faculty of Health Sciences, Queen's University, Kingston, Ontario, Canada; 5 Center of Excellence in Retina, Faculty of Medicine, Chulalongkorn University, Bangkok, Bangkok, 10330, Thailand

**Keywords:** visual impairment, depression, systematic review, meta-analysis, protocol

## Abstract

**Background:**

Disability is widely acknowledged as a major contributor to depression, which is a prevalent mental health condition that profoundly affects individuals, especially those with visual impairments.

**Objectives:**

This develops a protocol for a systematic review and meta-analysis aim to examine the prevalence and incidence of depression among visually impaired people globally.

**Methods:**

This systematic review and meta-analysis will be conducted in accordance with the Preferred Reporting Items for Systematic Reviews and Meta-Analyses (PRISMA-P) guidelines. A thorough search will be conducted using the PubMed, MEDLINE, EMBASE, and ProQuest databases to identify relevant studies published between January 1, 2011, and August 31, 2024, without limitations on language. Two out of three independent reviewers will screen the abstracts and full-text articles, extract data, and assess the risk of bias. Heterogeneity among the included studies will be evaluated individually. Meta-analysis of the pooled prevalence and incidence estimates will be conducted using the metaprop function in StataBE 18.0. Publication bias will be assessed through funnel plot analysis and Egger’s test.

**Conclusion:**

This protocol defines the planned scope and methodology for a forthcoming systematic review and meta-analysis, which aims to provide updated evidence on the prevalence and incidence of depression among individuals with visual impairment. Accordingly, it will facilitate a systematic review and meta-analysis to address the existing research gap by providing precise estimates of the pooled prevalence and incidence of depression in this population. The findings will offer valuable insights to inform public health policy, advocate for further research, and underscore the critical need to prioritize the mental health concerns of individuals with visual impairment.

**Systematic review registration:**

The protocol has been registered on the Open Science Framework (OSF) Registries with the Registration DOI: 10.17605/OSF.IO/6J4Y2.

## Introduction

Visual impairment is the third leading cause of disability globally. As of 2020, approximately 1.1 billion individuals worldwide have been affected by some degree of visual impairment. When categorized by severity, an estimated 295 million people (3.5% of the global population) experience moderate to severe vision impairment, 258 million (3.3%) have mild vision impairment, 510 million (6.5%) have near vision impairment, and 43 million (0.5%) are blind. Notably, at least 771 million cases (90%) of visual impairment globally are preventable or treatable. ^1^

Eye health refers to the enhancement of vision, ocular well-being, and functional ability, all of which contribute to overall health, well-being, social inclusion, and quality of life. It is a vital component in achieving several Sustainable Development Goals (SDGs). Conversely, compromised eye health and vision impairment adversely affect quality of life and hinder equitable access to education and employment opportunities. ^2^

In addition, it is linked to decreased functional capacity, greater disability, a higher risk of falls, social isolation, institutionalization, and increased mortality. The global economic burden of visual impairment exceeds US$3 trillion annually, impacting patients, their families, and communities. Beyond its direct effects on vision, visual impairment also impedes societal development and progress, affecting both local communities and the broader population. ^3^

Visual impairment can have significant impacts on physical health, quality of life, and mental well-being. A recent systematic review found that vision impairment serves as indicators of frailty in community-based studies, with depression being a major associated condition. ^4^

Depression is notably prevalent among individuals with visual impairment. According to a recent study by the Centers for Disease Control and Prevention (CDC), approximately one in four adults with visual impairment reported experiencing depression. Moreover, younger adults with visual impairment were found to have nearly five times the risk of developing severe depression compared to older adults. ^5^ However, prevalence estimates vary widely, ranging from 14% to 44%, depending on the specific population studied.

Depression constitutes a prevalent mental health disorder, characterized by a sustained state of low mood or emotional despondency, accompanied by anhedonia or a diminished capacity to derive pleasure from previously enjoyable activities. It is further typified by pervasive feelings of guilt or worthlessness, a marked decline in energy levels, difficulty concentrating, changes in appetite, psychomotor slowing or restlessness, sleep disturbances, persistent suicidal thoughts, recurrent over a protracted period. ^6^

According to a report by the World Health Organization, an estimated 322 million individuals globally are impacted by depression, representing roughly 4.4% of the world’s population. The prevalence of this pervasive mental disorder is increasing, with a particularly pronounced rise observed in low- and middle-income countries. ^7^ Depression can significantly impact various aspects of an individual’s well-being, including interpersonal relationships, academic performance, and occupational functioning. ^6^

Several studies have indicated a strong association between visual impairment and depression, with individuals having vision impairment being more vulnerable to developing depressive symptoms. ^8– 11^ Though, the global epidemiology of depression among individuals with visual impairment remains limited, with variations in prevalence and incidence estimates across different populations and study methodologies.

This systematic review and meta-analysis protocol aims to assess the global prevalence and incidence of depression among individuals with visual impairment. By systematically reviewing and quantitatively synthesizing existing studies, this review will enhance understanding of the mental health burden associated with visual impairment, inform public health initiatives, and guide future research and interventions for this at-risk population.

### Protocol

## Methods

### Study registration

Our research will involve a systematic review and meta-analysis protocol, adhering to the PRISMA-P guidelines. ^12^ The protocol for this review has been registered on the Open Science Framework (OSF) Registries with the Registration DOI: 10.17605/OSF.IO/6J4Y2.

### Eligibility criteria for study selection

Studies will be included if they meet the following criteria: (1) peer-reviewed articles published between January 1, 2011, and August 31, 2024; (2) studies involving individuals with visual impairment; and (3) studies reporting the estimated prevalence of depression or providing sufficient data to calculate the prevalence or incidence of depression. Studies will be excluded if they are reviews, meta-analyses, commentaries, brief reports, or protocols, or if they involve ineligible populations or outcomes.

### Literature sources and search strategy

PubMed, MEDLINE, EMBASE, and ProQuest databases will be searched for relevant literature from January 1, 2011 to August 31, 2024. The search terms encompassed the utilization of relevant keywords, and synonymous words as follows: visual impairment (visual* or vision* or “poor vis*” or “low vis*” or blind*) AND depression (depress* or sadness or unhappy or “mood disorder”) AND prevalence or incidence (prevalen* or inciden* or rate or epidemiolog* or proportion).

### Study selection process

**Figure 1.**
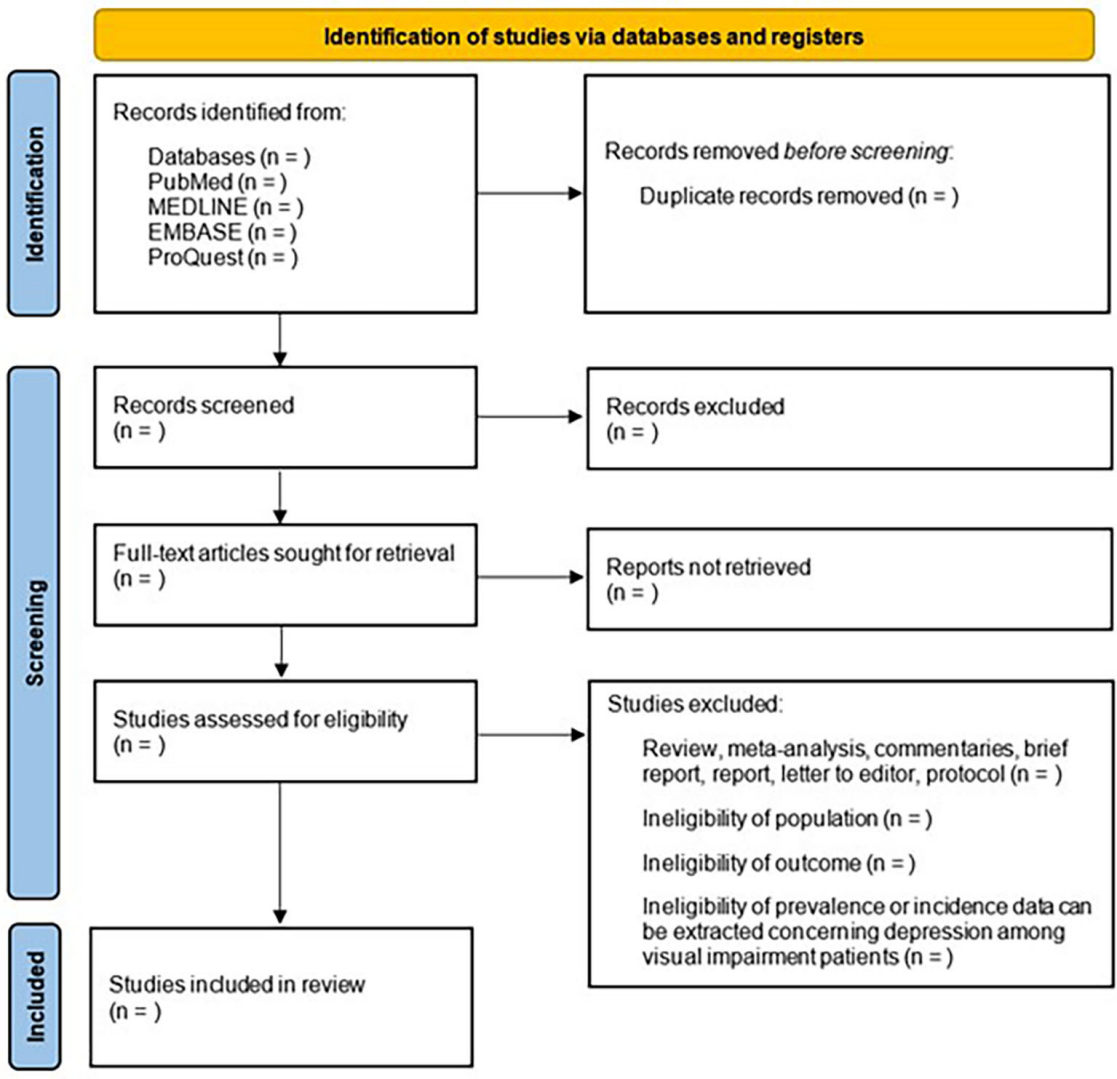
PRISMA 2020 flowchart of study selection.

The studies retrieved from the respective databases will be imported into Covidence software to streamline the management and organization of the screening process. Two reviewers will independently screen the titles and abstracts of all identified studies. Furthermore, the reviewers will independently evaluate the full texts based on the established eligibility criteria. Any disagreements will be resolved through consensus with the involvement of a third reviewer. The PRISMA 2020 flowchart of study selection is shown in Figure 1.

### Data extraction

Two reviewers will independently extract data using a predefined data extraction form. The extracted data will include study details (e.g., author(s), publication year, study period, study design, country, and study setting), participant characteristics (e.g., sample size, age, type of visual impairment assessment, and level of visual impairment), and outcome measures (e.g., depression assessment tool, number of depression cases among individuals with visual impairment, total number of individuals with visual impairment, and prevalence or incidence of depression). Any discrepancies encountered during the data extraction process will be resolved through discussion, and if consensus cannot be reached, a third reviewer will be consulted to resolve the issue. These are demonstrated in Tables 1, 2.

**Table 1.** Characteristics of included studies.

Author	Published year	Study period	Study design	Country	Setting	Sample size	Age group	n	N
									
									
									

Note: n = Number of Visual Impairment with Depression Cases; N = Total Number of Visual Impairment Cases.

**Table 2.** Measurement tools of included studies.

Author	Depression tool	VI assessment	VI level	n	N
					
					
					

Note: VI = Visual impairment; n = Number of Visual Impairment with Depression Cases; N = Total Number of Visual Impairment Cases.

### Risk of bias assessment

The methodological quality and risk of bias in each study will be assessed using the Joanna Briggs Institute (JBI) critical appraisal tools for prevalence and incidence studies in meta-analysis. ^13^ The risk of bias will be categorized as low, moderate, or high. ^14^ To maintain consistency in quality assessment, any discrepancies between the two initial reviewers will be resolved through collaborative review of the study. If necessary, a third reviewer will be involved to make the final determination of the appraisal scores.

### Data synthesis and analysis

The metaprop function in StataBE 18.0 will be utilized to perform a meta-analysis of the pooled prevalence and incidence estimates. This function utilizes the Freeman-Tukey double-arcsine transformation to stabilize the variances, thereby normalizing the data before pooling. A random-effects Restricted Maximum Likelihood Estimation (REML) model will be employed to account for heterogeneity between studies. ^15^ The statistic was used to quantify inconsistency and evaluate the heterogeneity of the results. Values of 25%, 50%, and 75% were considered low, moderate, and high respectively, with values higher than 50% indicating significant heterogeneity. ^16^

Subgroup analysis will be performed based on possible potential sources such as study period, design, country, continent, income classification, setting (clinic vs community based), age group, VI assessment, VI level, and depression tool. A sensitivity analysis will be conducted using the Leave-one-out methodology, in which one study will be sequentially excluded, and the analysis results will be recalculated with the remaining studies included. Additionally, studies suspected to be sources of heterogeneity will be excluded as part of the sensitivity analysis. ^17^ Funnel plots and Egger’s test for asymmetry will be employed to evaluate publication bias in the meta-analysis that will be conducted in this study.

## Discussion

Individuals with visual impairments are often at a substantially higher risk of developing depression, with research showing that the prevalence of depressive symptoms is notably greater in this population in comparison to individuals without visual impairments. This heightened vulnerability may be attributed to various factors, including the challenges associated with daily functioning, social isolation, and the psychological impact of diminished independence. ^18, 19^

Previous research has examined the presence of depressive symptoms in individuals with visual impairment. While some studies have specifically emphasized clinical eye examinations, others have used self-reported measures to assess visual impairment. Importantly, the prevalence of depression in this population has been investigated using various measures of depressive symptoms, resulting in differing findings across studies. ^20– 24^

Furthermore, a previous systematic review included 10 studies conducted exclusively on elderly populations. However, due to the limitations of these reviews, a meta-analysis could not be conducted. ^25^ Previous systematic reviews and meta-analyses examined the prevalence of depression in individuals with visual impairment targeted community-dwelling adults, ^26^ while another focused on patients receiving care at eye clinics. ^27^

Moreover, no previous systematic review or meta-analysis has specifically investigated the incidence of depression in individuals with visual impairment, pointing to a significant gap in the literature. Thus, we identified an opportunity to undertake a systematic review and meta-analysis to evaluate the prevalence and incidence of depression in individuals with visual impairment. Our study intended to address the existing research gap by providing precise and reliable estimates of the pooled prevalence and incidence of depression within this specific population. Additionally, we aimed to assess the prevalence of depression among subgroups within the visual impairment population, considering factors such as study period, design, continent, income classification, study setting, age groups, methods of visual impairment assessment, severity of visual impairment, and tools used for depression assessment.

The strength of this review lies in its methodological rigor, incorporating subgroup analyses to elucidate variations in depression prevalence and identify critical determinants of mental health outcomes in individuals with visual impairment. The findings will contribute valuable insights to the field of public health and clinical practice by highlighting the burden of depression in this vulnerable population and informing targeted interventions, policies, and future research directions. Ultimately, this systematic review will enhance our understanding of the complex interplay between visual impairment and mental health, offering evidence-based guidance for healthcare providers, policymakers, and researchers to improve the psychological well-being of individuals living with visual impairment worldwide.

### Ethics and dissemination

Ethical approval for this systematic review and meta-analysis protocol was obtained from the Research Ethics Review Committee for Research Involving Human Research Participants, Group I, Chulalongkorn University, with approval number COA 126/68 and dated May 12, 2025. This protocol will be submitted for publication in a peer-reviewed journal. Upon completion, the results of the systematic review and meta-analysis will be disseminated through a subsequent peer-reviewed publication.

### Study status

The search and screening of articles have been completed. The synthesis and manuscript preparation are currently in progress.

## Author contributions

Tantirattanakulchai, Pankaew: Conceptualization, Data Curation, Investigation, Methodology, Project Administration, Visualization, Writing – Original Draft Preparation, Writing – Review & Editing

Hounnaklang, Nuchanad: Conceptualization, Funding Acquisition, Methodology, Supervision, Writing – Original Draft Preparation, Writing – Review & Editing

Win, Nanda: Conceptualization, Data Curation, Writing – Original Draft Preparation

Tepjan, Suchon: Conceptualization, Data Curation

Pongsachareonnont, Pear Ferreira: Conceptualization, Methodology

### Reporting guidelines

Figshare: PRISMA-P checklist recommended items to address in a systematic review protocol: The Prevalence and Incidence of Depression among Visually Impaired People: A Systematic Review and Meta-Analysis Protocol, https://doi.org/10.6084/m9.figshare.29135558.v1. ^28^

Data are available under the terms of the Creative Commons Attribution 4.0 International license (CC-BY 4.0).

## Data Availability

No data are associated with this article.
