# Supplementary material for: The Prevalence and Incidence of Depression among Visually Impaired People: A Systematic Review and Meta-Analysis Protocol
Source: F1000Res. 2025 Jul 4;14:654. [Version 1] doi: 10.12688/f1000research.166020.1 (PMC12402763; doi:10.12688/f1000research.166020.1)
Supplement: Peer review history

# Reviewer report: Reviewer response for version 1

**DOI:** 10.5256/f1000research.182857.r397401

**Published:** 2025-09-01

**Authors:** Ugochukwu Anthony Eze

**Affiliations:** 1 Department of Ophthalmology, Federal Medical Centre, Asaba, Nigeria

I am happy with the protocol of this study.

 The 1st sentence of the introduction needs a citation it is a very factual statement. 

 Where authors were relating vision to SDG. I think it's important to expand a bit. Read IAPB documents you will see that 11 of the 17 SDGs are directly or indirectly related to eye care. 

 Also, there is absolutely no need to get ethical approval to register a systematic review. The OSF registration or Prospero registration suffices.

 The protocol is very well written

Is the study design appropriate for the research question?

Yes

Is the rationale for, and objectives of, the study clearly described?

Yes

Are sufficient details of the methods provided to allow replication by others?

Yes

Are the datasets clearly presented in a useable and accessible format?

Not applicable

Reviewer Expertise:

Eye care, Ophthalmology especially public health aspect, Low Vision, Health Economics

I confirm that I have read this submission and believe that I have an appropriate level of expertise to confirm that it is of an acceptable scientific standard.

# Reviewer report: Reviewer response for version 1

**DOI:** 10.5256/f1000research.182857.r407916

**Published:** 2025-08-30

**Authors:** Soumen Karmakar

**Affiliations:** 1 Neuropsychiatry, Institute of Neurosciences Kolkata, Kolkata, West Bengal, India; 2 Raiganj Government Medical College and Hospital, Raiganj, India

This study protocol addresses an important gap in the literature: the global prevalence and incidence of depression among visually impaired individuals. The rationale is well-founded, given the established psychosocial burden of visual impairment and the variability in reported depression rates. The stated objective—to provide pooled prevalence and incidence estimates through systematic review and meta-analysis—is clear and clinically relevant. The study design, a systematic review and meta-analysis adhering to PRISMA-P, is entirely appropriate for the research question. The methodology is described in sufficient detail, including eligibility criteria, databases searched, time frame, screening strategy, bias assessment tools, and planned statistical methods (metaprop in Stata with subgroup and sensitivity analyses). This level of detail should allow replication by other researchers. Datasets are not yet available, as this is a protocol, but the planned data extraction tables are clearly structured and accessible. Overall, the protocol is methodologically rigorous and transparent, with strong potential to yield meaningful results.

Is the study design appropriate for the research question?

Yes

Is the rationale for, and objectives of, the study clearly described?

Yes

Are sufficient details of the methods provided to allow replication by others?

Yes

Are the datasets clearly presented in a useable and accessible format?

Yes

Reviewer Expertise:

Familiarity with psychiatric epidemiology and systematic reviews

I confirm that I have read this submission and believe that I have an appropriate level of expertise to confirm that it is of an acceptable scientific standard.
